# Climate sensitivity of *Cryptomeria japonica* in two contrasting environments: Perspectives from QTL mapping

**DOI:** 10.1371/journal.pone.0228278

**Published:** 2020-01-28

**Authors:** Hideki Mori, Kana Yamashita, Shin-Taro Saiki, Asako Matsumoto, Tokuko Ujino-Ihara

**Affiliations:** Forestry and Forest Products Research Institute, Tsukuba, Ibaraki, Japan; Aristotle University of Thessaloniki, GREECE

## Abstract

Long-lived forest tree species experience a wide range of environmental conditions throughout their lifespan. Evaluation of the underlying growth and development mechanisms of these species is essential to predict tree growth under climate change. This study investigated climate sensitivity to temperature, precipitation, dry periods, and the associated genomic regions in *Cryptomeria japonica*, Japan’s most commercially important tree. We used tree rings and common garden experiments with three clonal replicates planted in two contrasting environments in Kyushu (Kumamoto site) and Honshu (Chiba site), Japan. Tree growth showed a significant negative correlation with the dry period (>4 days) in March of the year of tree-ring formation at the Chiba site. In contrast, temperature and precipitation had little influence on tree growth. Quantitative trait locus (QTL) analysis was performed to investigate climate sensitivity to dry periods at the Chiba site, revealing 13 significant QTLs. One QTL showed a substantially large contribution to the overall climate sensitivity, accounting for 12.4% of the total phenotypic variation. The phenotypic variance explained (PVE) by other QTLs ranged from 0.9% to 2.9%, and the total PVE by all QTLs was 35.6%. These findings indicate that the tree population at the Chiba site could be vulnerable to drought in early spring and that the QTL showing the greatest impact on climate sensitivity may be closely related to genes associated with tolerance or adaptation to drought stress. The QTLs identified in this study could be useful for molecular breeding, forest management, and predicting the growth of *C*. *japonica* under a changing climate.

## 1. Introduction

Longed-lived forest tree species, with their limited dispersal ability, experience various environmental changes throughout their lifespan. As extreme climatic events such as droughts and heatwaves are expected to increase as a result of ongoing climate change [1], evaluating the underlying genetic mechanisms that control growth and development of these species under changing climatic conditions is essential to understand the local adaptation abilities of tree species to predict tree growth, which is necessary for the effective forest management and breeding programs [2,3].

The growth responses of tree species to climate changes have been evaluated by multiple studies [4–6]. These studies often targeted tree species adapted to dry or extremely cold climates (e.g., *Pinus*, *Larix*), such as temperate and boreal forests, and successfully identified climatic variables affecting tree growth. Although the number of studies conducted outside those climatic regions is rapidly increasing [7–10], climate sensitivity of tree species adapted to warm and humid climate remains relatively understudied, probably due to their weak growth response to interannual climatic variation. However, the impacts of climate change (e.g., droughts and heatwaves) are expected to rapidly increase globally [1], and the negative impacts of such events (e.g., seasonal drought; [2]) on tree species in humid climates are also likely to increase. Evaluating the mechanisms underlying these species’ growth response to varying climatic conditions changes is necessary to predict the impact of climate change in these forest ecosystems.

Previously, empirical research of climatic factors was limited on tree growth and consisted of dendrochronological studies, often using mature or old-growth trees to investigate the impact of climate variation over a large time scale (>100 years; [11–13]). In contrast, short-term climatic variation (e.g., decades) may also have a significant impact on tree growth, particularly during the initial growth phase (from seedlings to young trees; [14–17]). It is during this initial stage of growth that trees exhibit the largest response to environmental conditions [18]. Additionally, the initial growth of trees has been studied as an important trait in tree breeding [19–21]. Evaluating climatic factors which affect initial tree growth is important not only for the fundamental knowledge on growth response of tree species but also for forest management and molecular breeding under changing climate.

Phenotypic traits such as wood growth and properties are influenced by environmental and genetic factors [22]. Therefore, investigating the underlying genetic mechanisms of tree growth response to climate change is essential to gain a comprehensive understanding of climate sensitivity of tree species [23]. In this context, genetic analyses such as quantitative trait locus (QTL) mapping and association mapping have been conducted on many forest species to identify important genome regions (QTLs) and genes that influence complex traits, primarily growth (stem growth and bud phenology), wood properties (wood density and chemistry) [24], and climate sensitivity (i.e., growth response to climate change; [17]). Identification of the important genome region or genes in terms of climate sensitivity of tree species would be useful for predicting the performance of genotypes under climate change (genomic selection; [25,26]).

*Cryptomeria japonica* (sugi) is a wind-pollinated, allogamous, evergreen conifer species [27]. Sugi is the most widely planted and commercialized tree in Japan, with its straight trunk and soft wood, and it has many diverse uses, such as in house construction, ship building, and pulp production [28]. Vegetative propagation of *C*. *japonica* is easily conducted by cutting shoots, thereby allowing the replication of clones in common garden experiments across multiple environments. A large number of genetic markers have been developed for *C*. *japonica* [29,30], which allows the identification of QTLs strongly associated with genes. These characteristics make *C*. *japonica* suitable for the evaluation of QTLs associated with climate sensitivity.

Several studies have evaluated genomic regions that affect the growth and wood properties of *C*. *japonica* [30–32]. Long-term growth patterns of *C*. *japonica* have been evaluated using tree rings [33,34] and other methods (e.g., dendrometer; [35,36]). However, few studies have assessed the genetic mechanisms of growth patterns linked with climatic variability in *C*. *japonica*. In this study, we aimed to evaluate the growth patterns during temporal variations in temperature, precipitation, and dry period using tree rings (climate sensitivity traits; Housset et al. 2018); we also attempted to identify the genomic regions (QTLs) influencing climate sensitivity in *C*. *japonica* using common garden experiments in two contrasting environments in Japan.

## 2. Materials and methods

### 2.1. Plant material, common garden experiment, linkage map

The plant material, common garden experiment, and linkage map previously described by [32] were used in this study. The *C*. *japonica* population used in this study comprised 139 genotypes, which were obtained from crossing between two F_1_ progenies: YI96 (female parent) and YI38 (male parent). YI96 and YI38 were obtained by crossing local varieties in the Kyushu region of Japan, ‘Yabukuguri’ × ‘Iwao’ and ‘Yabukuguri’ × ‘Kumotooshi’, respectively [37,38]. Common garden experiments were established for the 139 genotypes with three clonal replicates in two different environments in Japan: the Chiba site (35°20′52.55′′N, 140°1′47.92′′E) and the Kumamoto site (32°41′58.36′′N, 130°45′17.64′′E). Trees at both sites were planted in 2005 and were harvested for phenotypic measurement in 2015 (Kumamoto) and 2016 (Chiba). The Kumamoto site in the Kyushu region represents the original climatic condition of the mapping progeny, which was derived from crossing local varieties of this region. We used two linkage maps based on 858 genetic markers (YI96: heterozygous; YI38: homozygous) to construct the YI96 linkage map and 916 genetic markers (YI38: heterozygous; YI96: homozygous) to construct the YI38 linkage map. Genetic markers with large numbers of missing values (>1%) were excluded. The average distance between adjacent markers in the YI96 and YI38 maps was 1.47 and 1.56 cM, respectively.

### 2.2. Tree-ring data

Wooden boards with a width of approximately 4 cm were placed 80 cm above the ground from the tree trunk of all trees in the sampled populations. All wooden boards included both pit and bark and were used to acquire scanned images using a conventional scanner. For each tree, two rectangular sections containing both pit and bark were selected from both sides of the scanned image. The selected sections were then used to measure tree-ring width. We averaged the width between two pairs of tree rings for each tree, which were then converted into basal area increment (BAI) chronologies. To control for the effects of age- and size-related growth, BAI chronologies were detrended using a modified Hugershoff model [39]. An autoregressive model (ar1) was used to reduce the temporal autocorrelation in the BAI chronologies, and the residuals were extracted (hereafter, relative BAI; S1 Fig). For each clone, Tukey’s biweight robust mean was calculated to obtain annual averages of the relative BAI. The above analyses were performed using R version 3.6.0 [40] using R packages “measuRing” [41] and “dplR” [42].

### 2.3. Climate data

Climate data (i.e., temperature and precipitation) of the two study sites during the study period were sourced from AMeDAS (Automated Meteorological Data Acquisition System of the Japan Meteorological Agency; https://www.jma.go.jp/jma/en/Activities/amedas/amedas.html). At the Chiba and Kumamoto sites, the mean annual temperature is 15.7°C and 16.4°C, respectively, and the mean annual precipitation is 1614.6 mm and 2285.5 mm, respectively. Both sites are characterized by contrasting climatic conditions in the growing season, particularly precipitation patterns. For instance, Kumamoto receives on average 2.5-fold more rainfall than Chiba during the growing season (May to August; [32]). We examined the effect of continuous dry (non-raining) days (hereafter, dry period) obtained from the precipitation data for each month (S2 Fig). The magnitude of drought stress on sampled populations can change with the length of the dry period [i.e., short dry periods (<1 day) may have no effect on tree growth]; thus, dry periods were accumulated for each month with varying thresholds, ranging from 1 to 7 days. As setting a higher threshold of dry days produces a larger ratio of zero data (i.e., raining days) per month, the largest threshold was set as 7 days to make the ratio of zero data less than 50% (S3 Fig) to avoid inaccurate estimation of climate sensitivity (described below). Climate data described above were linearly detrended prior to the analysis.

### 2.4. Climate sensitivity traits

Based on the methods of [17], climate sensitivity traits were obtained by calculating correlation coefficients between climate data (i.e., temperature, precipitation, and dry period) and relative BAI scores. Monthly temperature, precipitation, and dry period during the experimental years were used for the climate sensitivity traits. Correlation coefficients were calculated for the months starting from June of the year prior to tree-ring formation until September of the year of tree-ring formation.

### 2.5. QTL analysis

For each study site, QTL analyses of climate sensitivity traits in the sampled population were conducted using the Bayesian Lasso linear regression model:

y=μI+Xβ+e

where ***y*** is climate sensitivity traits, *μ* is the overall mean, ***I*** is the identity matrix, **X** is the genotype of each marker, ***β*** is the effect of the genetic marker on climate sensitivity traits, and ***e*** is the error. Heterozygous and homozygous marker genotypes were converted to 0 and 1, respectively. The Bayesian Lasso model computation was performed using the Empirical Bayesian Lasso algorithm (EBlasso; [43]) with three-level hierarchical prior distribution (normal, exponential, and gamma prior; [44]):

$$\beta_{i}∼N(0,\sigma_{i}),\;(i=1,2,\ldots ,k)$$

$$\sigma_{i}^{2}∼\exp(\lambda ),$$

λ∼gamma(a,b)

where k is the number of genetic markers and *a* and *b* are regularization parameters that control the level of shrinkage of the marker effects (*β*) toward zero. The regularization parameters were determined using five-fold cross validation. The EBlasso was conducted using R package “EBglmnet” [44]. Genetic markers with non-zero effects and *P* < 0.01 were considered as significant QTLs. The phenotypic variance explained (PVE) by *i*-th QTL was estimated as ${PVE}_{i}={\hat{\beta }}_{i}^{2}\sigma_{x_{i}}/\sigma_{y}$, where $\hat{\beta }$ is the estimated value of parameter *β, σ_x_* is the variance of the marker genotype, and *σ_y_* is the variance of climate sensitivity traits. PVE by all QTLs was estimated as ${PVE}_{all}=\sigma_{\hat{y}}/\sigma_{y}$, where $\sigma_{\hat{y}}$ is the variance of the estimated value of the climate sensitivity traits, which is obtained from a multiple linear regression using significant QTLs as explanatory variables [45]. The prediction accuracy of significant QTLs was tested by correlating the observed and predicted values using five-fold cross validation. The results of the QTL analysis were validated with composite interval mapping (CIM) using R package “qtl” [46].

## 3. Results

### 3.1. Climate sensitivity traits

**Fig 1:**
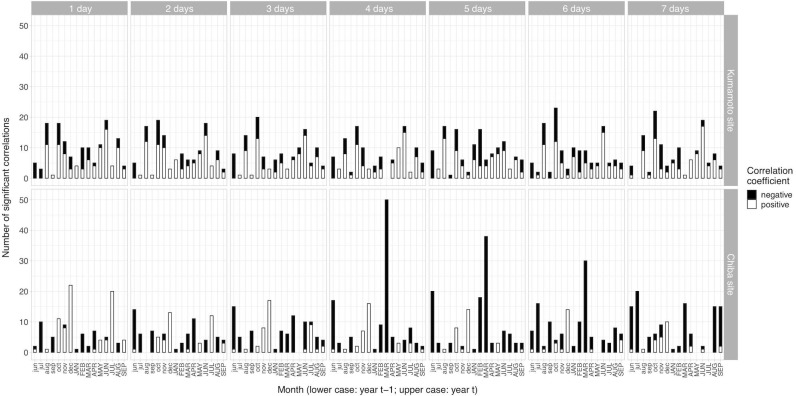
Climate sensitivity of growth to dry period at the two sites. Climate sensitivity in terms of significant correlation between growth and dry period at Kumamoto (upper panels) and Chiba (lower panels) sites are shown with varying thresholds (from 1 to 7 days). Panel columns represent the thresholds. Positive and negative correlations are shown with open and closed bars, respectively. Month with lower case represents the month of the year prior to tree-ring formation (from June to December), whereas month with upper case represents the month of the year of tree-ring formation (from January to September).

**Fig 2:**
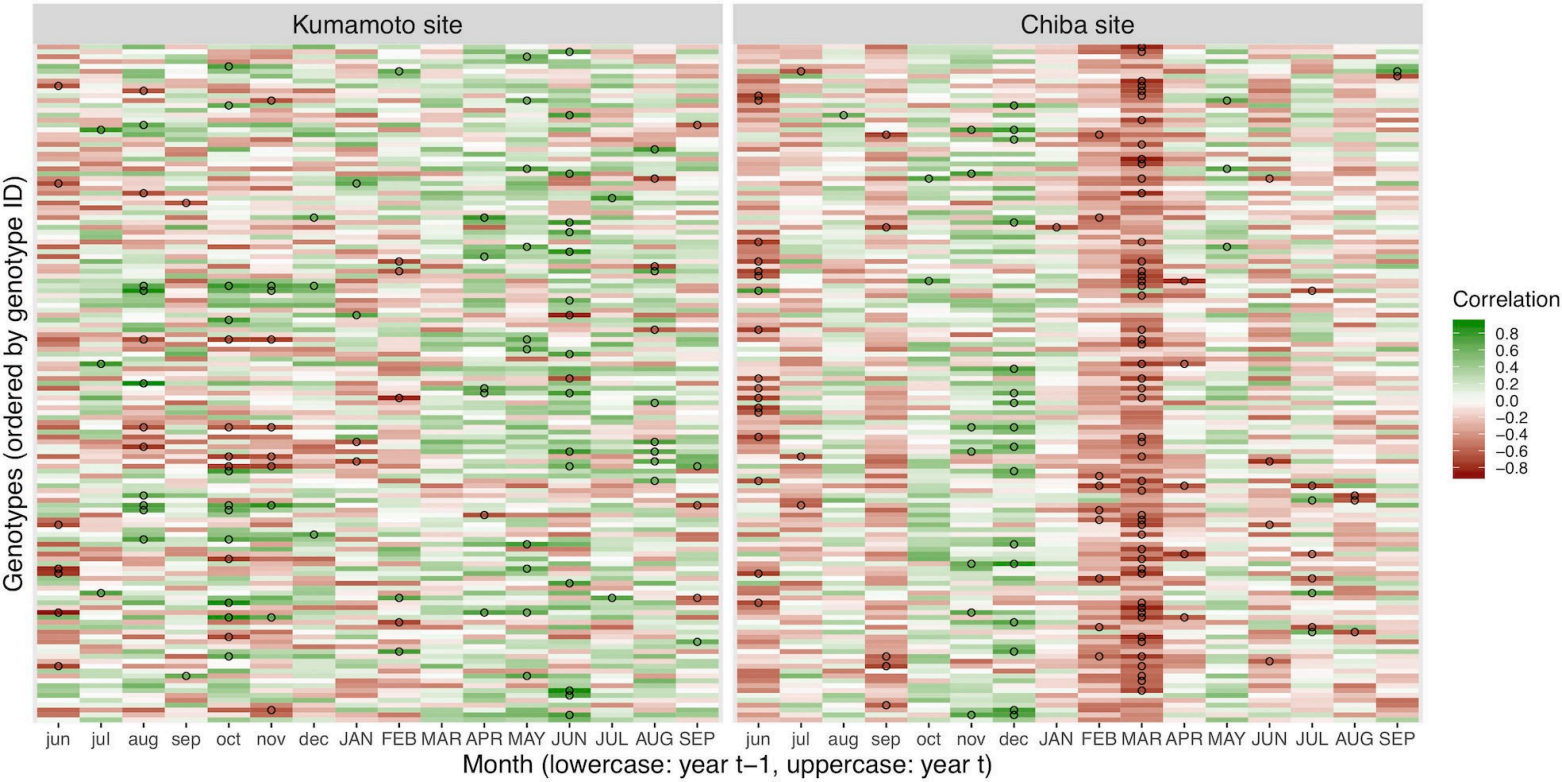
Heatmaps of growth response to dry period with a threshold of 4 days. The climate sensitivity traits of mapping populations at the Kumamoto (left panel) and Chiba (right panel) sites are shown as correlation coefficients between relative basal area increment and monthly cumulative dry period with a threshold of 4 days. Open circles indicate significant correlations (*P* < 0.05). Month notation is the same as in Fig 1.

Trees at the Chiba site exhibited a significant negative growth response to dry periods (with threshold of 4 days) in March of the year of tree-ring formation (Figs 1 and 2). This significant response to drought gradually decreased with larger thresholds (>5 days; Fig 1). On the other hand, trees at the Kumamoto site did not exhibit a strong growth response to dry periods (Figs 1 and 2) despite the fact that the number of dry days in both sites was not significantly different (S4 Fig). The maximum number of clones with significant correlations at the Kumamoto site was 23, which was recorded in October of the year of tree-ring formation, whereas the maximum number of clones with significant correlations at the Chiba site was 50, which was recorded in March of the year of tree-ring formation as mentioned above (Fig 1). The growth response to drought for the Chiba population at the site varied with the set threshold, whereas the corresponding response at the Kumamoto site appeared to depend on the genotypes (Figs 1 and 2).

In contrast, growth response to monthly temperature and precipitation were relatively weak for the mapping populations at both sites (S5 Fig). The maximum number of clones with significant correlations was 23 and 25 for temperature and precipitation, respectively, which were both found in July of the year prior to tree-ring formation at the Chiba site. Similar to the climate sensitivity to drought, the tree population at the Kumamoto site showed varying responses to temperature and precipitation, whereas the trees at the Chiba site showed positive or negative responses to temperature and precipitation (S5 Fig).

Because a significant drought response was found at the Chiba site (Figs 1 and 2), we proceeded with QTL analyses on the climate sensitivity traits (with threshold of 4 days) in March of the year of tree-ring formation at this site.

### 3.2. QTL analysis

**Fig 3:**
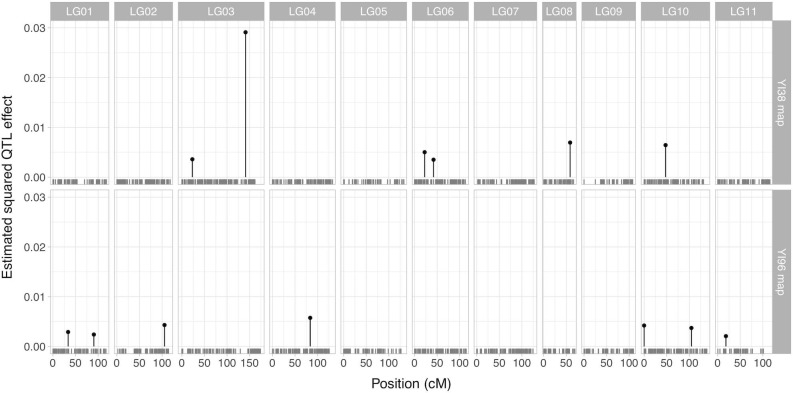
Effect and location of significant QTLs for drought sensitivity at the Chiba site. Panel columns represent linkage groups. Panel rows represent two types of linkage maps based on the marker types (upper: YI96 map; lower: YI38 map). Short vertical lines at the bottom of each panel represent marker positions.

**Fig 4:**
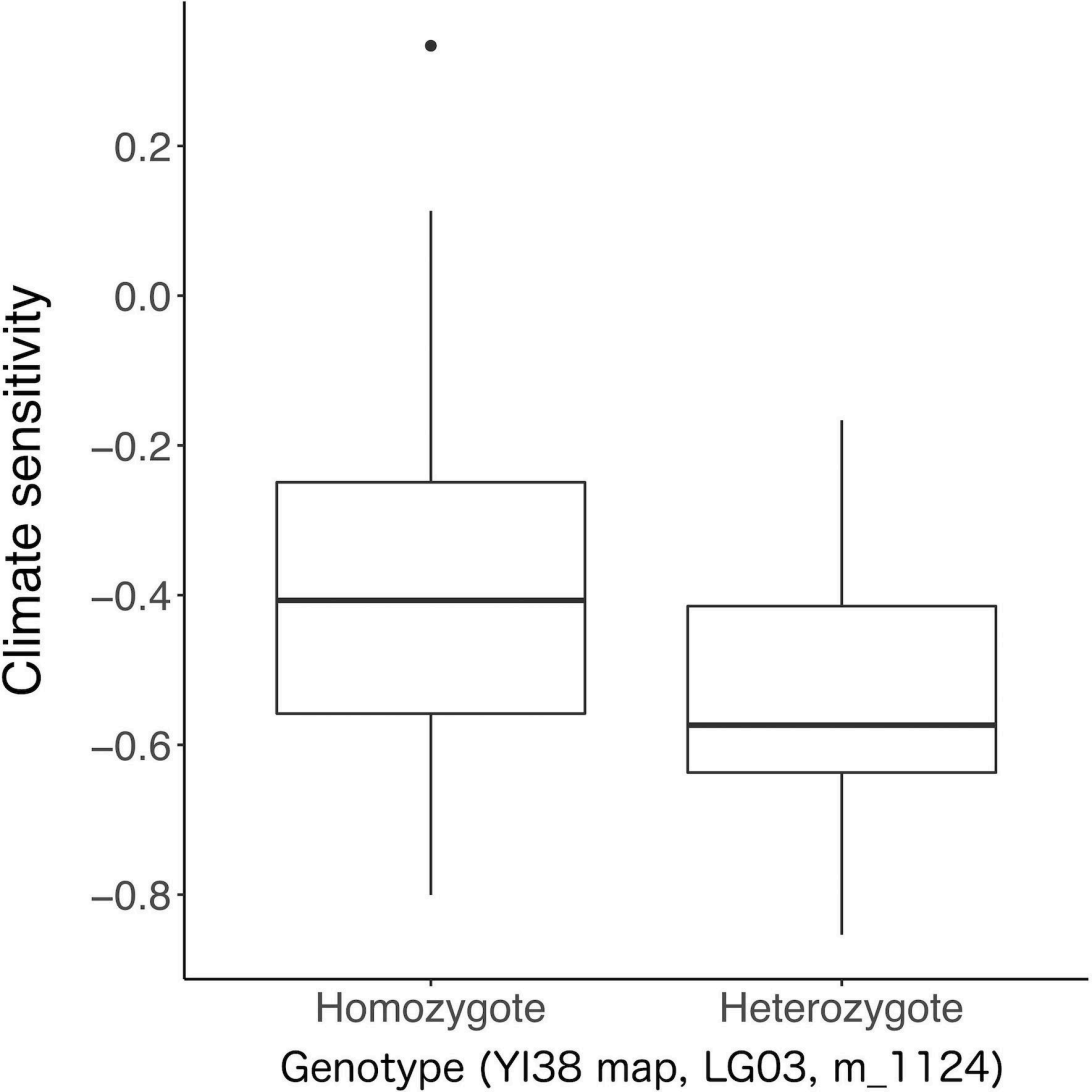
Difference in climate sensitivity between the marker genotype of the strongest QTL identified for drought sensitivity at the Chiba site.

QTL analysis on sensitivity to drought in March of the year of tree-ring formation for the Chiba trees revealed 13 significant QTLs (Fig 3; Table 1). Of these, one QTL located in linkage group 3 of the YI38 map had a significant effect compared with the remaining 12 QTLs (Figs 3 and 4; Table 1). The PVE of the strongest QTL was 12.4%, while that of other QTLs ranged from 0.9% to 2.9%. The PVE by all QTLs combined was 35.9%. Climate sensitivity differed by 0.14 in average between the marker genotype of the strongest QTL (Fig 4). A significant positive correlation (*r* = 0.57, *P* < 0.001) was found between the observed and predicted values of significant QTLs (S6 Fig). Identified QTLs were validated with CIM (S7 Fig); most were identified at similar positions in the linkage maps, and the logarithm of odds (LOD) score of the QTL with the largest contribution was 11.9.

**Table 1:** List of significant QTLs identified for drought sensitivity at the Chiba site.

Marker name	Linkage group	Linkage map	Position (cM)	Beta	PVE^a^ (%)
m_1124	LG03	YI38 map	140.943	−0.171	12.4
m_1534	LG08	YI38 map	58.521	−0.083	2.9
m_1640	LG10	YI38 map	47.554	−0.080	2.7
m_0325	LG04	YI96 map	83.624	−0.076	2.5
m_1331	LG06	YI38 map	22.273	0.071	2.1
m_0149	LG02	YI96 map	105.401	−0.066	1.8
m_0708	LG10	YI96 map	0.000	−0.065	1.8
m_0788	LG10	YI96 map	104.723	0.061	1.6
m_1034	LG03	YI38 map	23.201	−0.060	1.5
m_1343	LG06	YI38 map	41.999	0.059	1.5
m_0031	LG01	YI96 map	33.557	0.054	1.2
m_0063	LG01	YI96 map	90.525	0.049	1.0
m_0817	LG11	YI96 map	18.205	0.046	0.9

^a^ Phenotypic variance explained

## 4. Discussion

### 4.1. Climate change sensitivity

The growth response at the Chiba site indicates that those trees have a high sensitivity to drought stress during the early spring. This may be a result of the seasonal variation of gas exchange in *C*. *japonica*: [47] reported that whole plant evaporation increased significantly from March to April, possibly because this species requires a lot of water for gas exchange (i.e., photosynthesis) during early spring. High water demand during a dry period decreases the plant’s water potential; the water deficit further causes dehydration and cavitation in leaf and stem, resulting in cell turgor [48–50], which inhibits photosynthesis and metabolic activity [51–53]. The response of *C*. *japonica* stem growth to water deficit was investigated previously using irrigation experiments [54,55]. The negative growth response during dry periods in early spring is also consistent with the seasonal growth patterns of *C*. *japonica* [35,36,56,57]; studies reported that the stem diameter growth of *C*. *japonica* occurred mostly between April and June. [36] investigated the radial growth patterns of 16 *C*. *japonica* populations across Japan, and reported that trees started to grow from April to May, peaking until around June, and decreased to almost zero after June. This tendency was also reported in other studies [35,56,57], indicating that our observations may reflect the seasonality of both growth patterns and gas exchange traits related to water demand of *C*. *japonica*, suggesting that stem diameter growth is probably influenced by water availability during early spring.

On the other hand, the mapping populations at the Kumamoto site exhibited a significantly lower growth response than populations at the Chiba site although there was no significant difference in dry period between the two sites. Furthermore, the mapping population at the Chiba site tended to show either positive or negative growth in response to climatic factors, whereas the growth response of the mapping population in Kumamoto site was both positive and negative depending on the genotype. The differences observed at the two sites may be due to their transfer from their original habitat to a different environment, which is known to negatively impact tree growth (e.g., *Abies sachalinensis*, [58]; *Pinus contorta*, [59]). As mentioned earlier, the tree populations in this study were derived from a local variety from the Kyushu region, where the Kumamoto site is located. Indeed, decreased tree growth at the Chiba site compared with that at the Kumamoto site was also previously reported [32]; the study found that the mean stem diameter of trees at the Kumamoto site was larger than at the Chiba site despite the Kumamoto trees being 1 year younger. This indicates that Kumamoto is more suitable for tree growth and is probably more robust to drought conditions than the Chiba site, which may explain the contrasting growth patterns observed in our study. Another possible explanation might be that there was a significant difference in precipitation between the two sites during the growing season: the amount of rainfall from May to August was 2.5-fold higher in Kumamoto than in Chiba. The Chiba population therefore experienced more drought stress during the growing season than the Kumamoto population. This might have caused a delayed effect of drought stress in early spring at the Chiba site [60,61], which partly explains the lack of a significant growth response to drought at the Kumamoto site.

A relatively intense growth response to temperature and precipitation was found in July of the year prior to tree-ring formation at the Chiba site; however, the sensitivity to temperature and precipitation was relatively weak compared to sensitivity to drought conditions, as indicated by the number of significant correlations (i.e., temperature: 23; precipitation: 25; and dry period: 50). This indicates that climatic factors influencing the growth of *C*. *japonica* may be difficult to identify using monthly temperature and precipitation data alone. The lack of sensitivity to temperature can partially be explained by the wide distribution range of *C*. *japonica*, which is widely adapted to various environments. Natural forests of this species range from the Aomori prefecture (40°42′N, 140°12′E) to Yakushima Island (30°15′N, 130°30′E) [27,62]. As the study sites were spread over a wide latitudinal range (within the species’ natural distribution range), the temporal variation in temperature observed may not sufficiently influence tree growth. However, the observed lack of sensitivity to precipitation could be due to more extreme climatic events, such as a large amount of rainfall during a short period (e.g., typhoons). Such climatic events can significantly increase monthly precipitation but have a limited influence on water availability for tree growth. This indicates that water availability for the growth of *C*. *japonica* cannot be fully explained by monthly precipitation alone and highlights the importance of using other indicators more likely to reflect water availability and its influence on tree growth [63]. This may be particularly important for tree species adapted to humid climates because such species’ climate sensitivity is often difficult to assess compared with those adapted to drier habitat (e.g., *Pinus*, [5]; *Larix*, [4]).

### 4.2. Climate sensitivity QTLs

The QTL located in linkage group 3 in the YI38 map contributed significantly to the climate sensitivity of *C*. *japonica* (PVE = 12.4%). The PVE magnitude of the QTL is considered to be relatively high; in comparison, the generally reported effect of QTLs in other tree species is often less than 5% (reviewed in [24]). The large contribution of this QTL to climate sensitivity indicates that it plays a significant role in tree growth under drought conditions. Additionally, this QTL could be closely related to genes associated with growth response to drought stress. Key genes in drought tolerance and adaptation of plant model species (e.g., *Arabidopsis thaliana*, *Populus*) and other forest tree species (e.g., *Pinus*, *Picea*) have been reported in previous research (reviewed in [64]), and further research with similar approaches on *C*. *japonica* is essential to identify the same genes. Furthermore, identifying genes associated with drought stress is of importance for future forest management as climate change is expected to increase the frequency of extreme climatic events, such as drought and heatwaves [1].

Apart from the strongest QTL, there were a number of significant QTLs identified with a relatively small contribution to overall climate sensitivity (<3%). This implies that drought sensitivity in *C*. *japonica* is influenced by a number of genes, each with a different effect on the overall climate sensitivity phenotype. This observation was consistent with the findings of previous QTL mapping studies on the growth traits of major tree species (e.g., *Pinus taeda*, [65]; *Eucalyptus globulus*, [66]). These studies indicate that, similar to growth, climate sensitivity is also influenced by many QTLs with relatively small effect sizes. It is important to note that the contribution to climate sensitivity by all identified QTLs (35.9%) was almost three-fold larger than the strongest QTL alone (12.4%), and the intermediate prediction accuracy was found using identified QTLs (*r* = 0.57, *P* < 0.001). This indicates that the 13 QTLs identified in this study might be useful in predicting the drought sensitivity of *C*. *japonica*. Likewise, using genomic data to predict climate change sensitivity is important to inform future forest management practices, particularly for long-lived forest tree species.

## 5. Conclusions

This study found that dry periods (longer than 4 days) strongly influenced the growth response of *C*. *japonica* mapping population at the Chiba site. Moreover, we found one QTL with a large contribution to overall climate sensitivity, possibly related to a key gene associated with growth response to drought stress. In contrast, we did not find a significant growth response to drought stress at the Kumamoto site, which was the original habitat of the mapping progenies. This indicates that climate sensitivity of the study population is probably influenced by surrounding environmental conditions, which highlights the need for further research on climate sensitivity of *C*. *japonica* under various conditions. This includes research on life history stages, climate (i.e., experiments covering wider geographical locations), and other environmental conditions (e.g., soil conditions; [67]). The epigenetic effects on genotypes might also be important for the growth response observed in the present study [68, 69]. Additionally, because our study was conducted using local varieties from the Kyushu region, additional studies capturing wider genetic variation (e.g., populations from natural forests) with different approaches—such as genome-wide association study (GWAS)—are necessary to generalize the findings of this study. It is also important to note that a further study using the mapping progenies derived from the parents in different provenances (e.g. Honshu region), could provide significant insights into the growth response of *Cryptomeria japonica* found in the present study. Furthermore, it is possible that the climate adaptation QTLs have been fixed in the Kyushu region, and were monomorphic in the mapping progenies. This could be tested by crossing *C*. *japonica* trees in and outside the Kyushu region. Finally, it is important to note that, while heavy rainfall events (100 mm/day) are increasing, the number of rainfall days in Japan has decreased over the past 4 decades [70]. If this tendency continues in the future, the frequency of dry periods > 4 days (which had significant influence on tree growth at the Chiba site) may also increase. Although *C*. *japonica* is currently planted or naturally distributed in humid climates, the negative impact of climate change on the growth of *C*. *japonica* via short-term droughts may become more severe, which requires more research on the climate sensitivity of this species in the near future.

## Supporting information

S1 Fig Clonal averages of raw (A, B) and relative (C, D) basal area increment (BAI) at the Kumamoto site (A, C) and Chiba site (B, D).
(PDF)

S2 Fig Continuous dry (non-raining) days at (A) the Chiba site and (B) the Kumamoto site. The horizontal dashed line indicates the threshold of 4 days of dry period.
(PDF)

S3 Fig Varying thresholds and the ratio of zero data (no dry period in a given month). The horizontal dashed line represents 50% of the ratio of zero data.
(PDF)

S4 Fig Frequency of dry days in March of the year of tree-ring formation at (A) the Chiba site and (B) the Kumamoto site.
(PDF)

S5 Fig **Growth response to temperature (right panels) and precipitation (left panels) at the Kumamoto site (upper panels) and the Chiba site (lower panels).** Positive and negative correlations are shown with open and closed bars, respectively. Month notation is the same as in Fig 1.
(PDF)

S6 Fig Prediction accuracy of the 13 identified QTLs. (PDF)

S7 Fig Results of the QTL analysis using composite interval mapping. The horizontal dashed lines represent the logarithm of odds (LOD) scores of two and three.
(PDF)

S1 Table Tree ring width of the mapping populations at the two sites. (CSV)

S2 Table Marker genotypes and positions. (CSV)
