# Supplementary figures and images for: Climate sensitivity of Cryptomeria japonica in two contrasting environments: Perspectives from QTL mapping
Source: PLoS One. 2020 Jan 28;15(1):e0228278. doi: 10.1371/journal.pone.0228278 (PMC6986750; doi:10.1371/journal.pone.0228278)

**(A)**

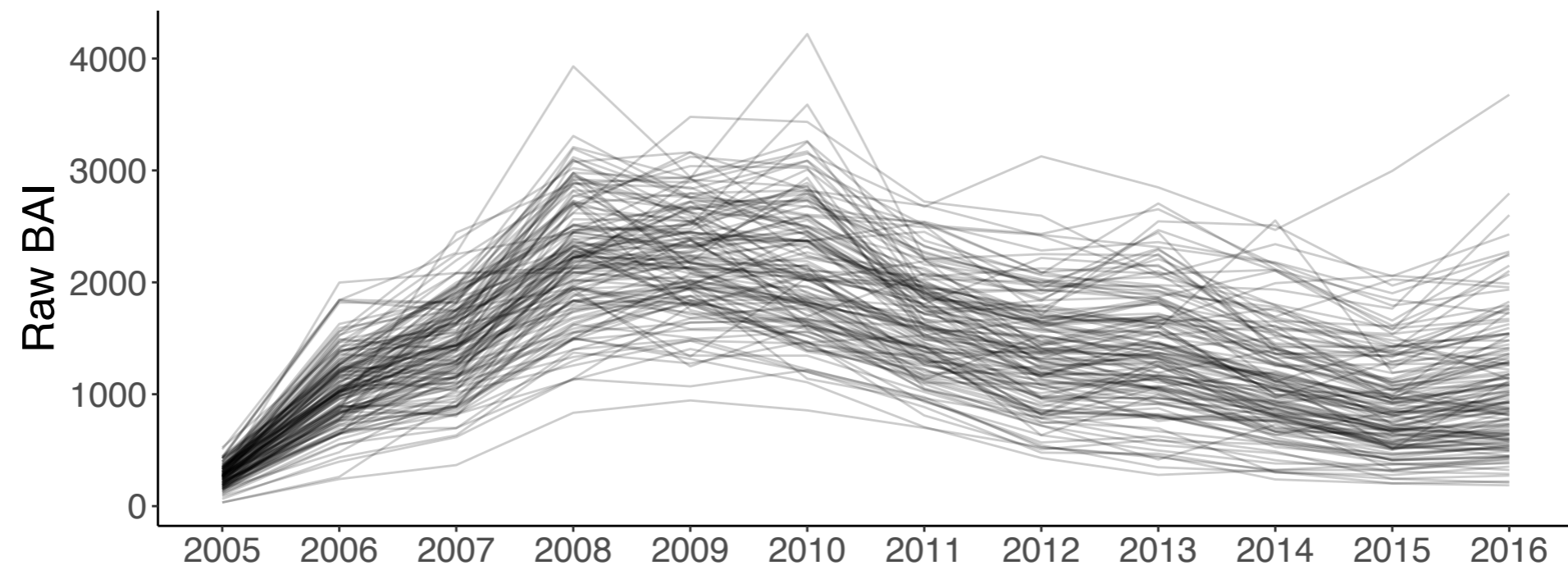

**(B)**

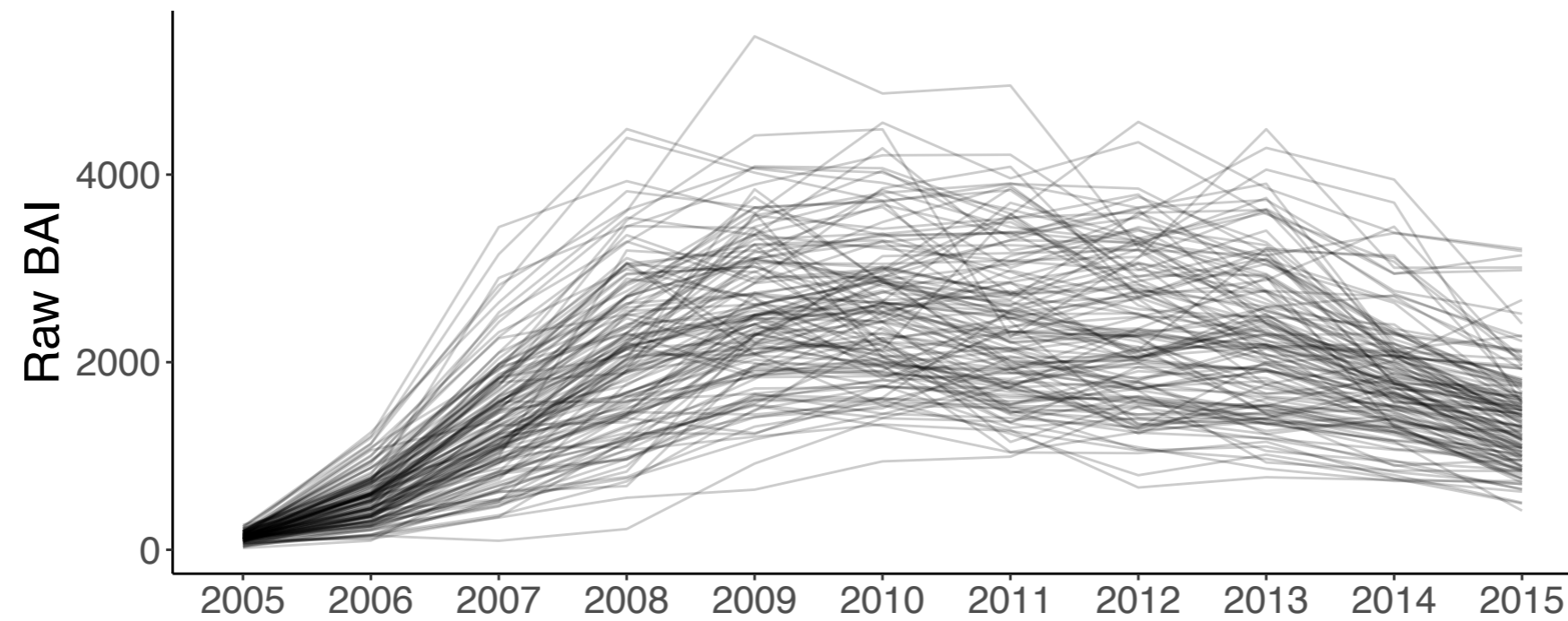

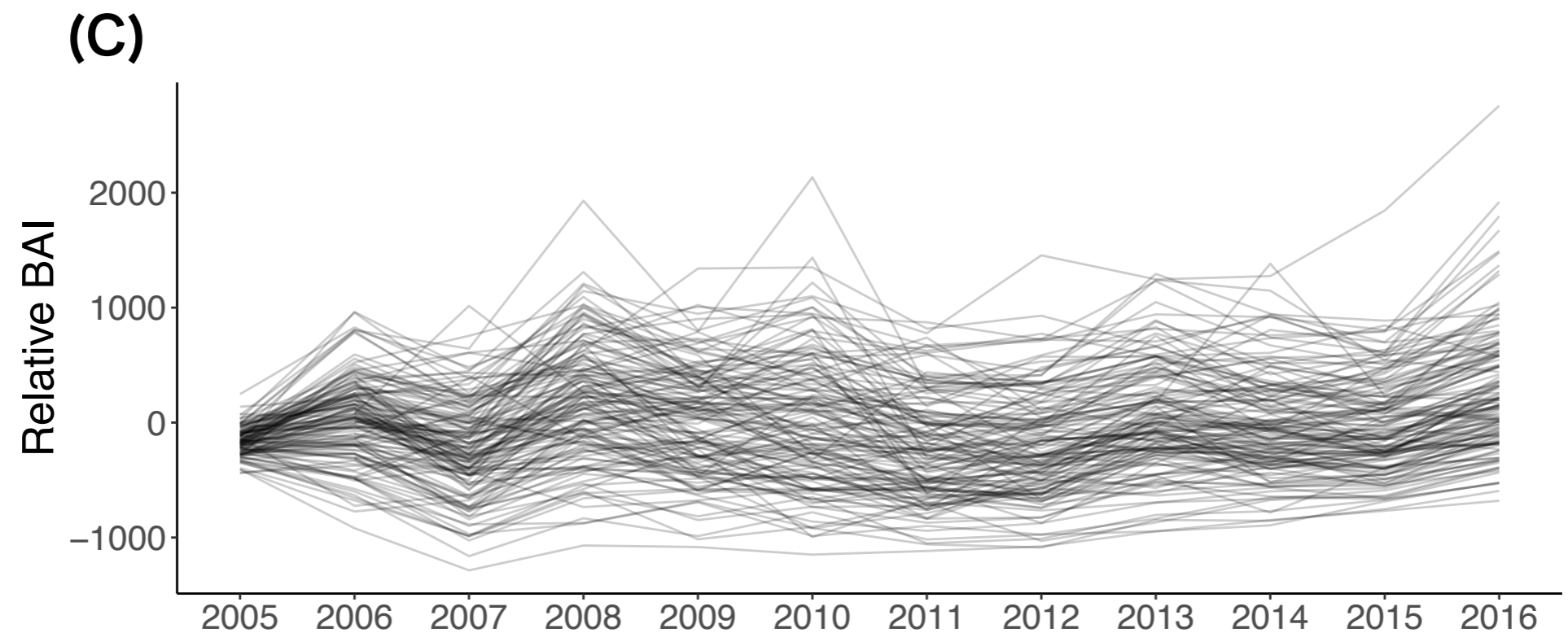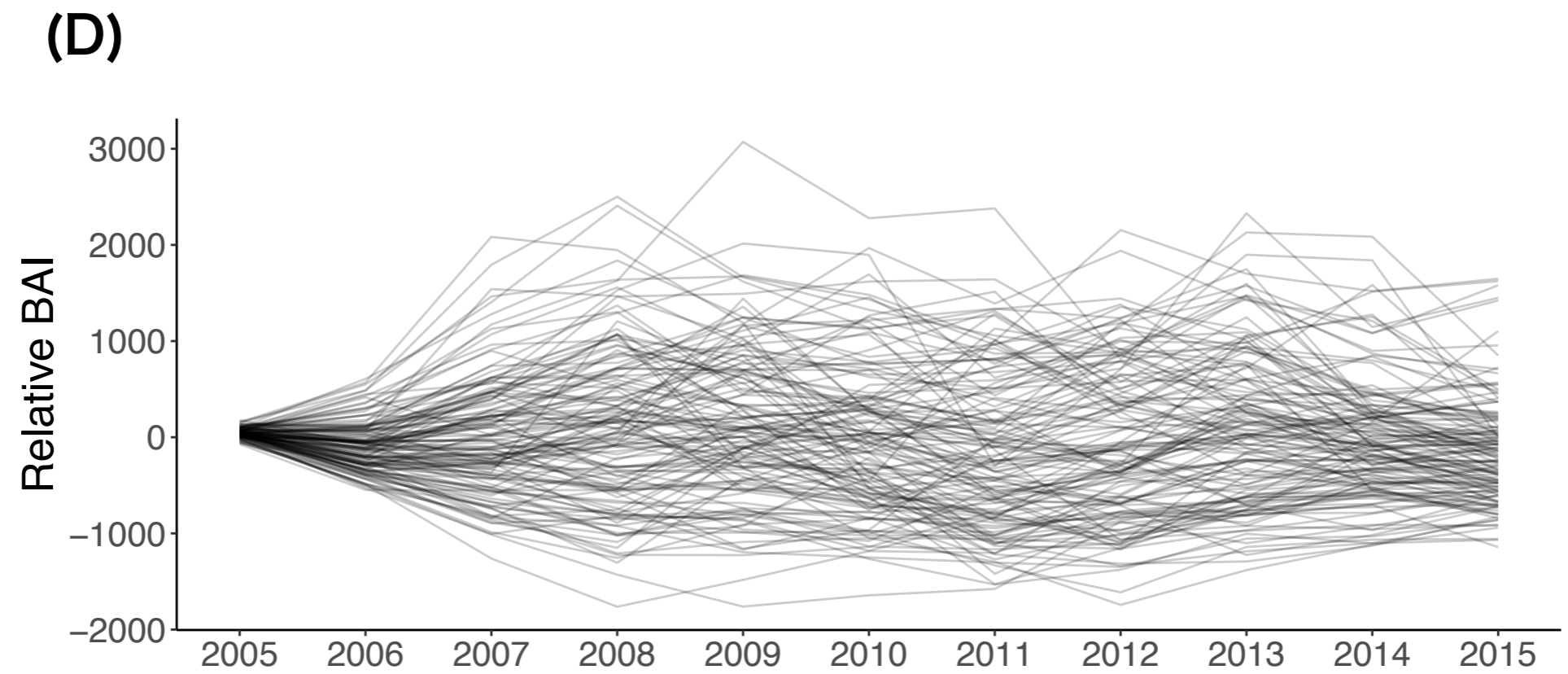

Supplement: S1 Fig — Clonal averages of raw (A, B) and relative (C, D) basal area increment (BAI) at the Kumamoto site (A, C) and Chiba site (B, D). (PDF) [file pone.0228278.s001.pdf]

(A)

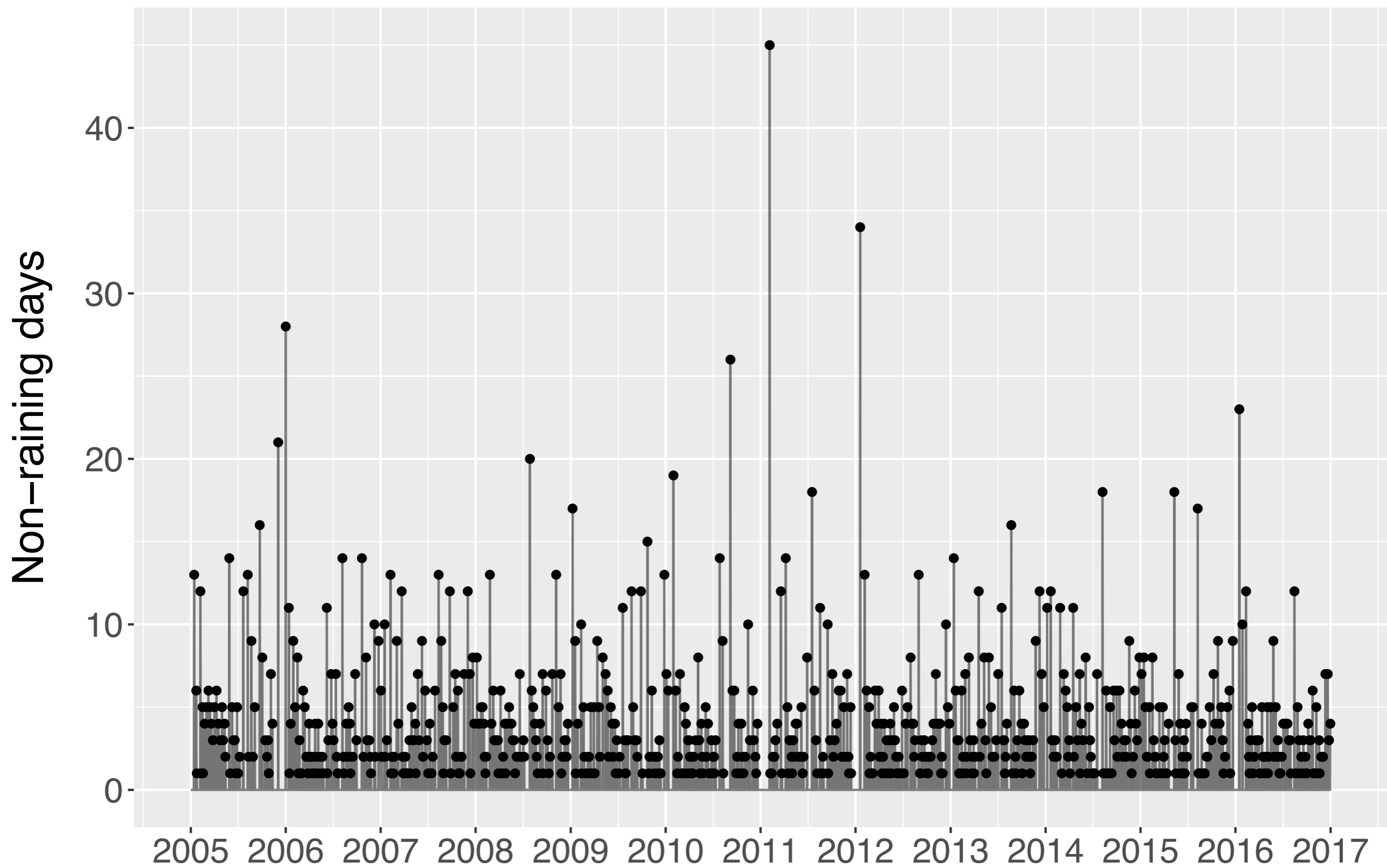

(B)

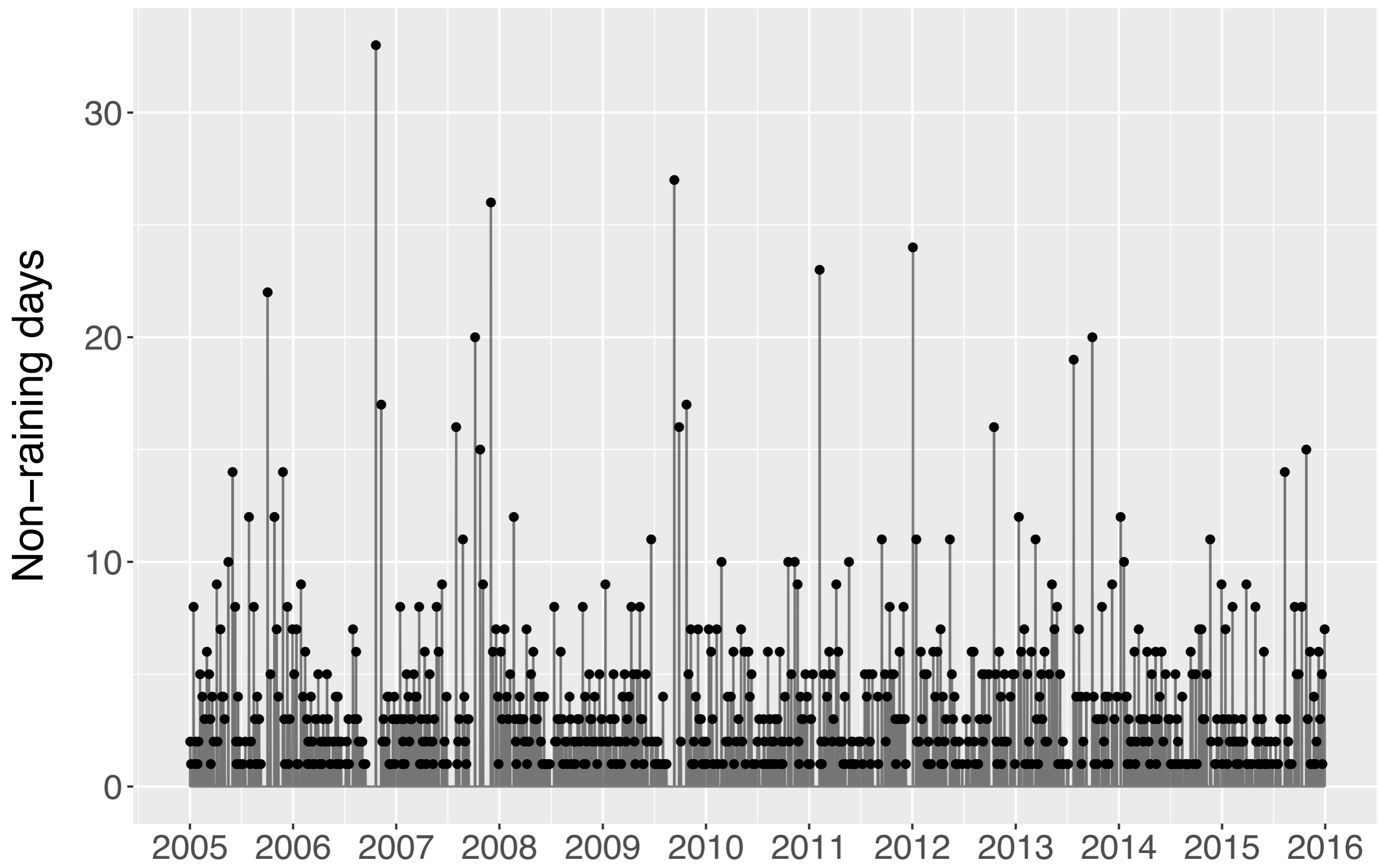

Supplement: S2 Fig — Continuous dry (non-raining) days at (A) the Chiba site and (B) the Kumamoto site. The horizontal dashed line indicates the threshold of 4 days of dry period. (PDF) [file pone.0228278.s002.pdf]

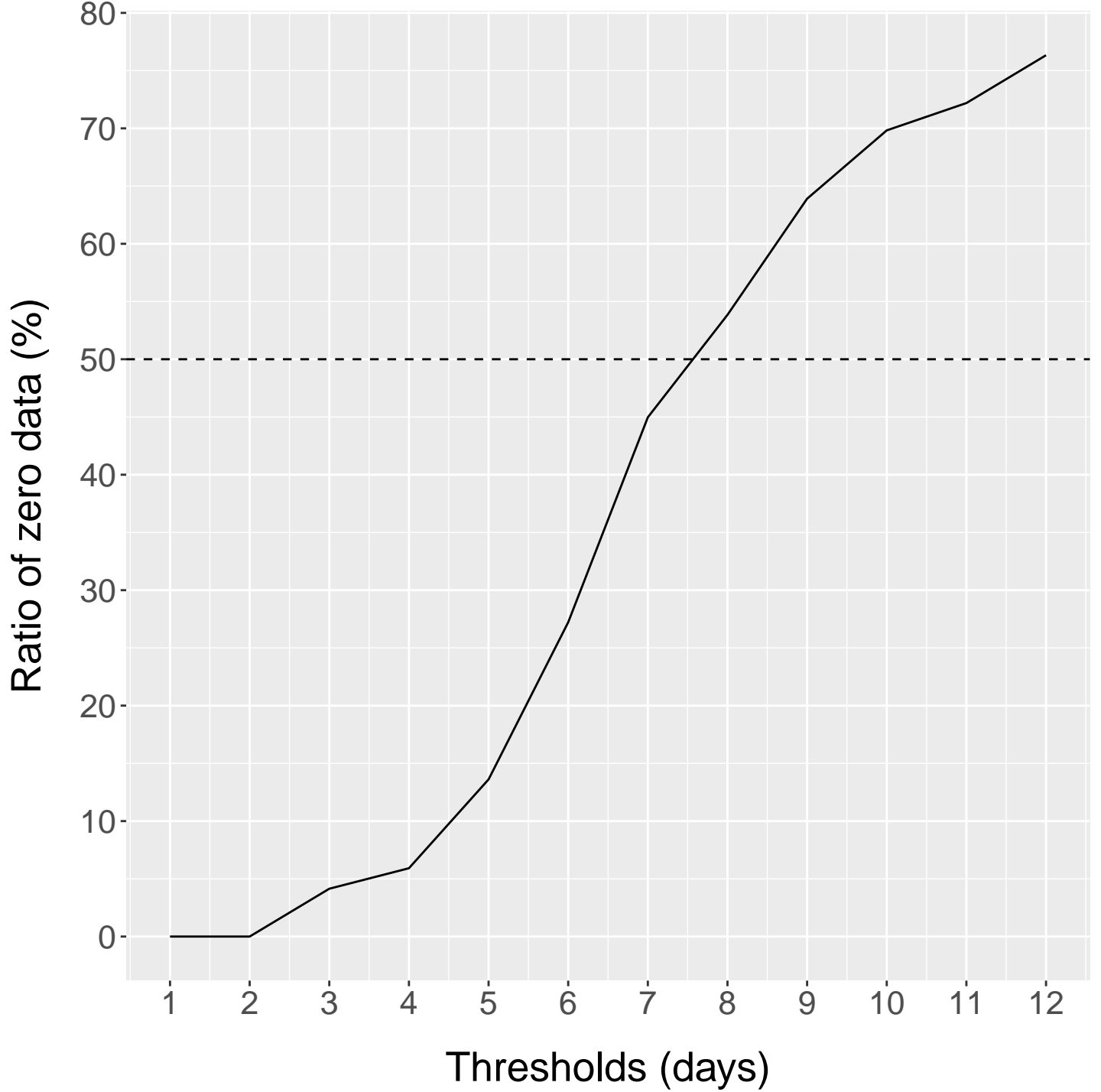

Supplement: S3 Fig — The horizontal dashed line represents 50% of the ratio of zero data. (PDF) [file pone.0228278.s003.pdf]

(a)

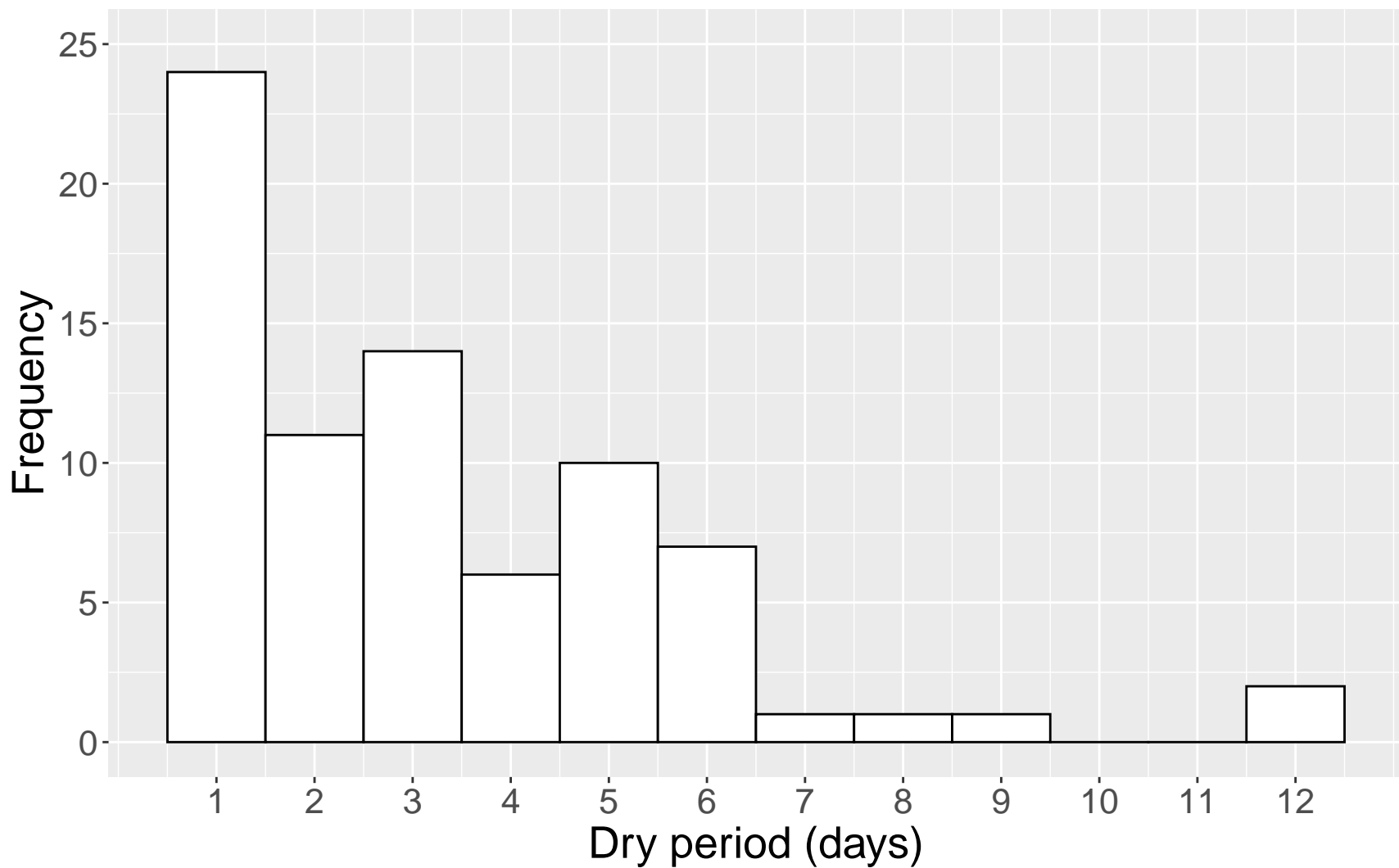

(b)

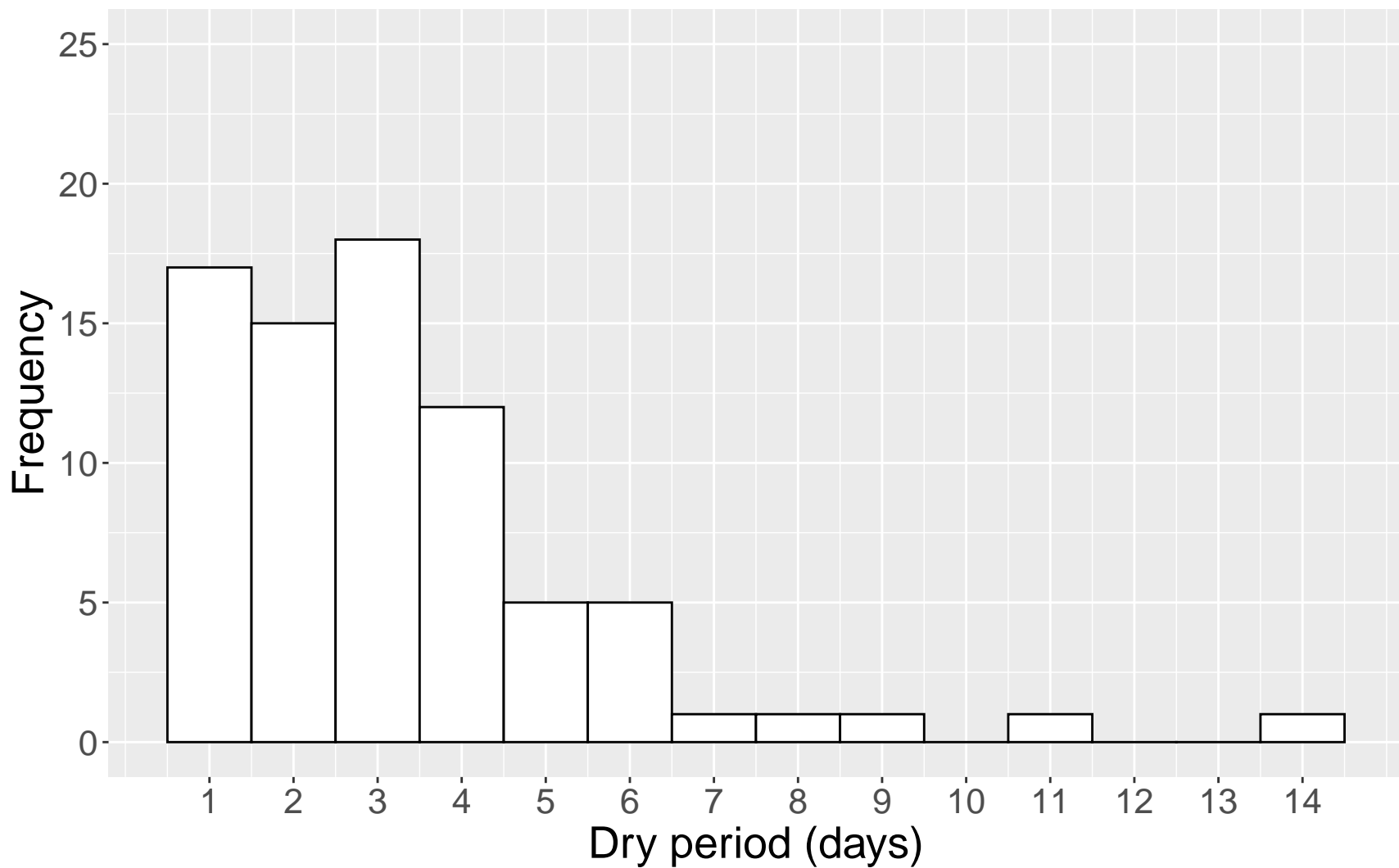

Supplement: S4 Fig — Frequency of dry days in March of the year of tree-ring formation at (A) the Chiba site and (B) the Kumamoto site. (PDF) [file pone.0228278.s004.pdf]

Number of significant correlations

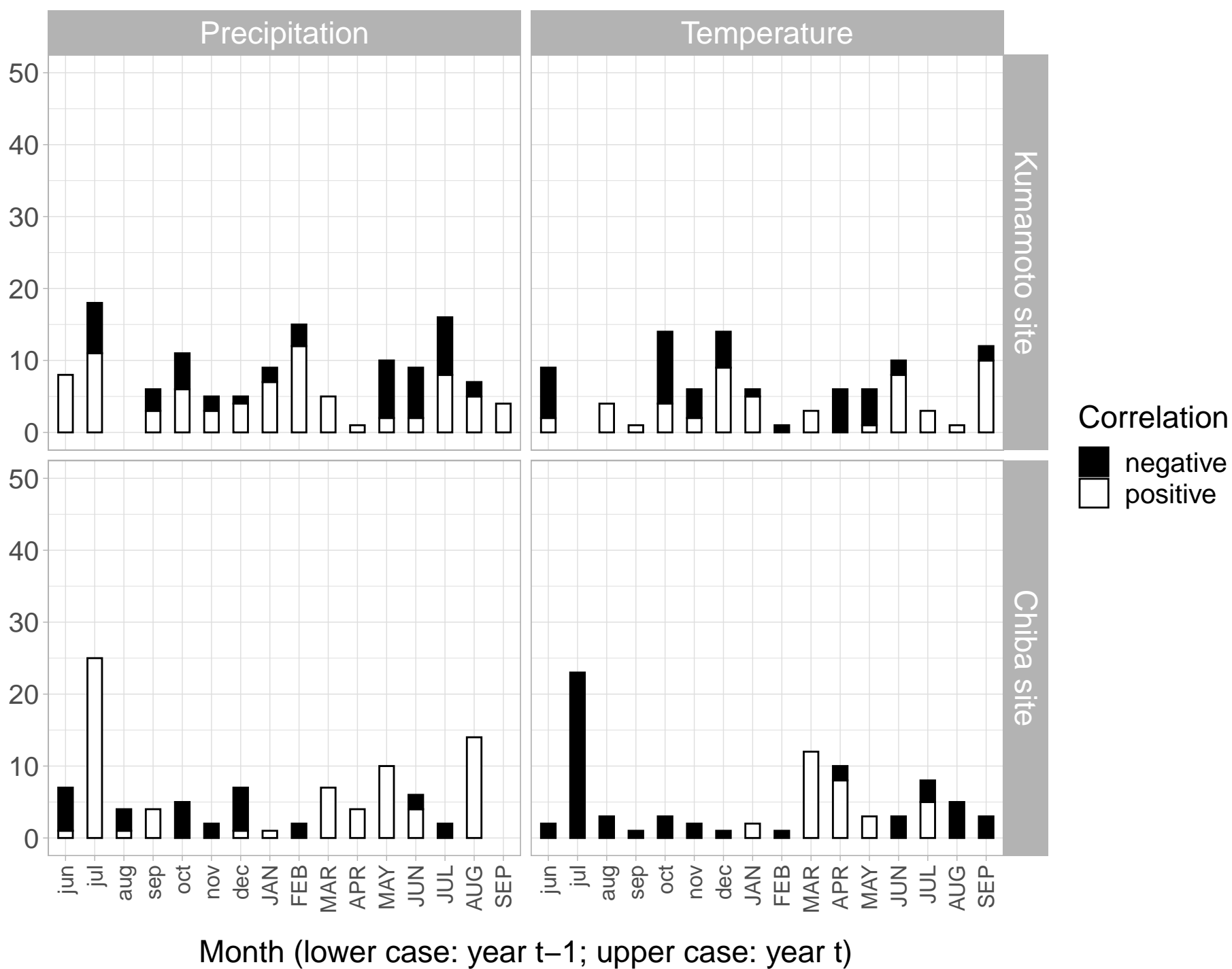

Supplement: S5 Fig — Growth response to temperature (right panels) and precipitation (left panels) at the Kumamoto site (upper panels) and the Chiba site (lower panels). Positive and negative correlations are shown with open and closed bars, respectively. Month notation is the same as in Fig 1. (PDF) [file pone.0228278.s005.pdf]

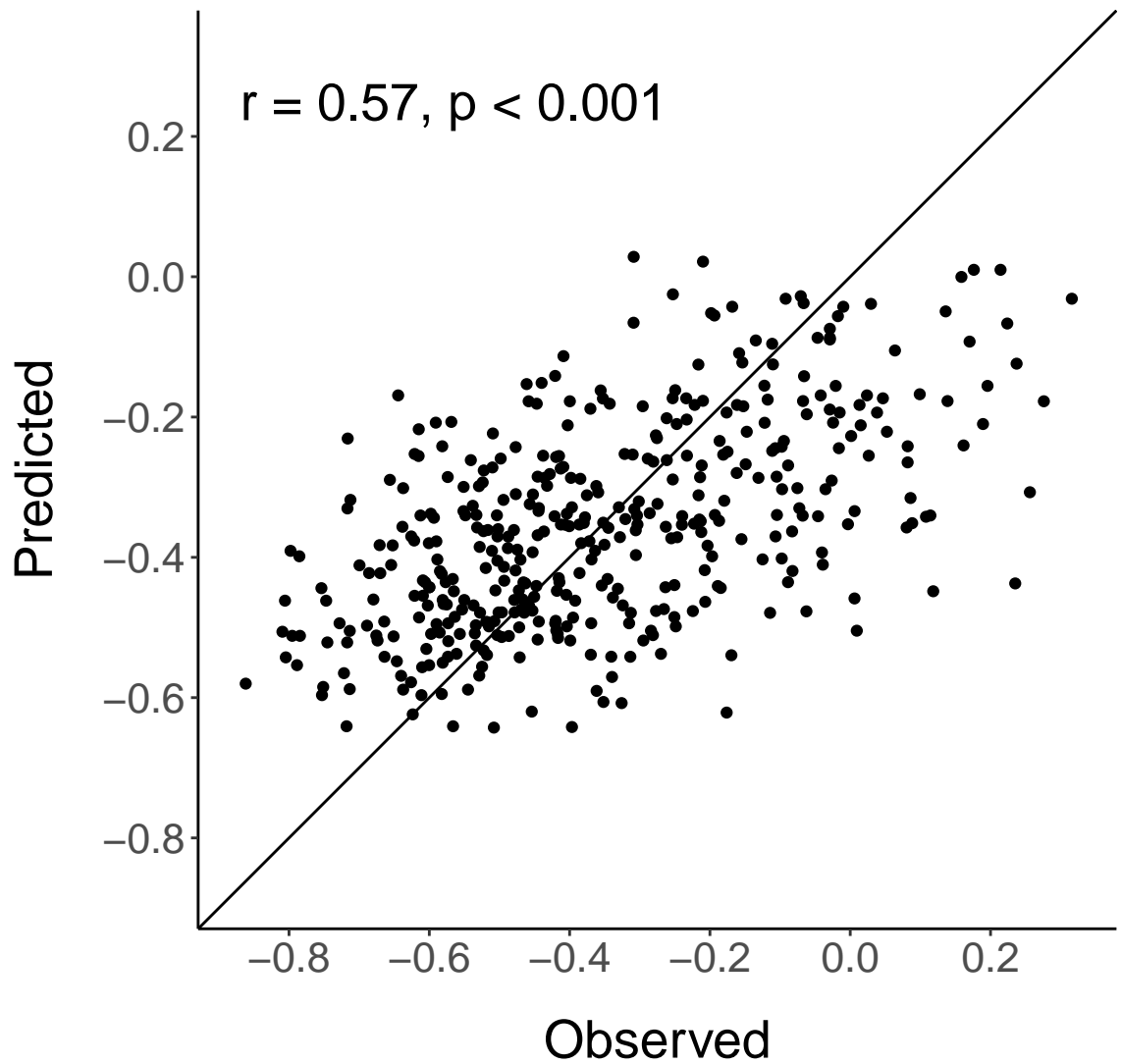

Supplement: S6 Fig — (PDF) [file pone.0228278.s006.pdf]

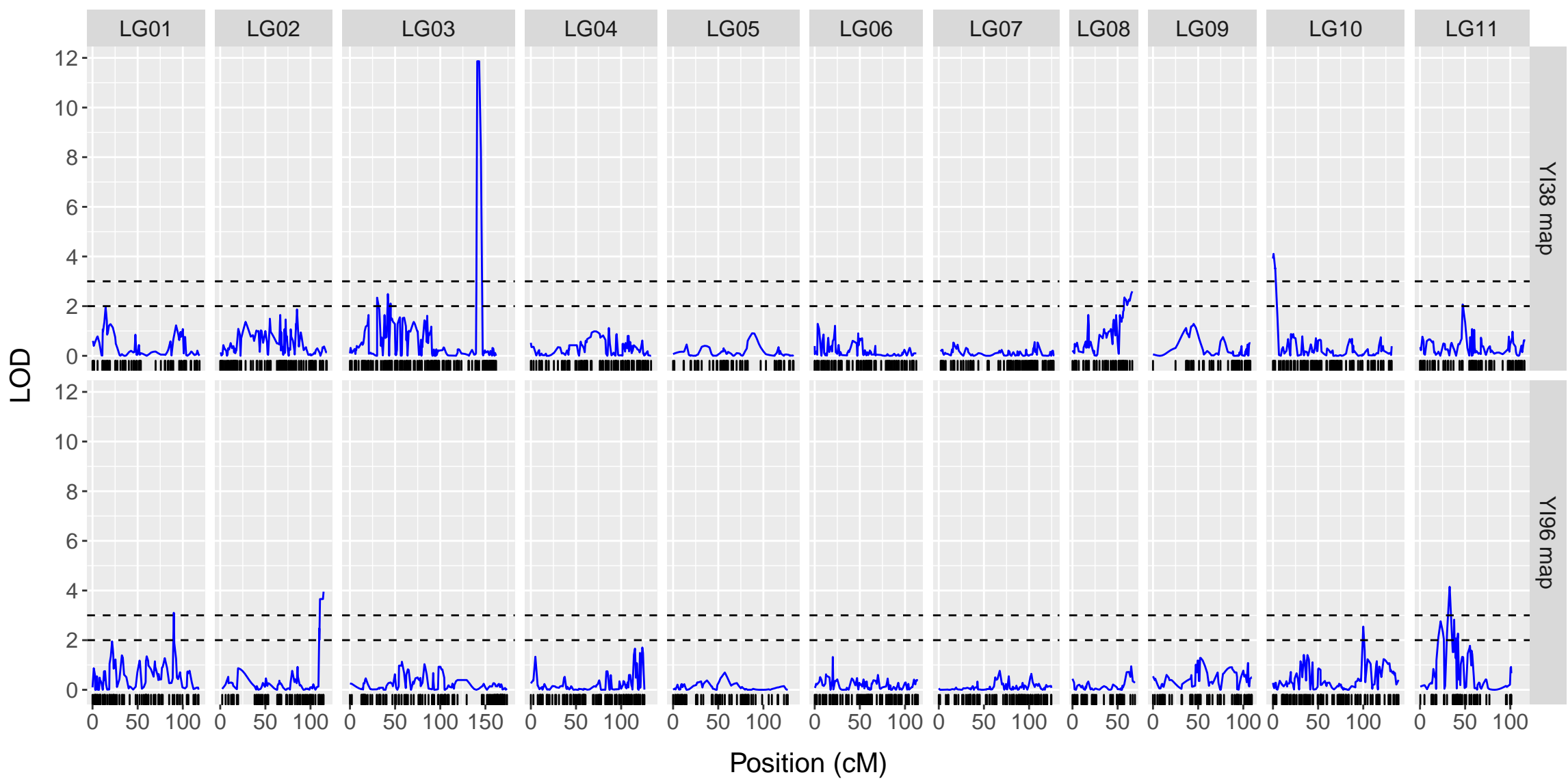

Supplement: S7 Fig — The horizontal dashed lines represent the logarithm of odds (LOD) scores of two and three. (PDF) [file pone.0228278.s007.pdf]
